# Pandora's Box

**DOI:** 10.1192/bji.2020.33

**Published:** 2020-08

## Of women and men

It is believed that men are more likely than women to die by suicide although women make more suicidal attempts. A recent meta-analysis/systematic review of nine studies and databases compared age-standardised suicide mortality ratios in the general population and in female and male physicians. They also examined whether there was a reduced ratio in the cohort of the year1980 compared with the years before.

The findings were: (a) women physicians had higher suicide mortality ratios than the general female population and male physicians; (b) male physicians had significantly lower suicide mortality ratios compared with the general male population; (c) both male and female physicians’ suicide mortality ratios had significantly decreased after 1980 versus before.

The good news is that the overall suicide mortality ratios are going down at least as far physicians are concerned but the female predominance remains a concern. Further research into these gender differences and the factors involved are needed.

Duarte D., El-Hagrassy M. M., Couto T. C. E.,  (2020). Male and female physician suicidality: a systematic review and meta-analysis. JAMA Psychiatry, 77: 587–973212981310.1001/jamapsychiatry.2020.0011PMC7057173.

## Stressful times

The effects of the COVID pandemic, racism and other major adversities are stressors with which we have been living for some time; but how do we cope? The subjective feelings of stress vary between individuals and some of us manage better than others.

Our subjective feelings of stress are not always in line with our physiological responses to stress such as the activation of the hypothalmo–pituitary–adrenal axis and the release of cortisol. Researchers from Yale University have recently demonstrated that the neural networks emanating from the hippocampus and their connectivity to other relevant areas of the brain have a major role to play in our subjective experience of stress.

They studied normal volunteers, in whom they carried out functional magnetic resonance imaging scans while being exposed to a barrage of highly aversive and threatening images. Their results showed distinct patterns of hippocampal connectivity, which predicted either enhanced or diminished feelings of stress. Enhanced subjective feelings of stress were associated with more active functional neural connectivity between the hippocampus and the hypothalamus whereas more active functional connectivity between the hippocampus and the lateral prefrontal cortex (the executive part of the brain) were associated with lower levels of feelings of stress.

The authors speculated that memory may play a role. It is known that hippocampal volume is associated with life stress and through its complex neural network it may contribute to the subjective feeling of stress by supporting memory retrieval, which depending on its content, may either increase or decrease stress responses.

Goldfarb E. V., Rosenberg M. D., Dongju Seo D.,  (2020) Hippocampal seed connectome-based modeling predicts the feeling of stress. Nature Communications, 11(1): 265010.1038/s41467-020-16492-2PMC725344532461583.

## How dogmatic can you be?

Pandora has discussed a study on entrenched beliefs and the underlying brain mechanisms involving emotional pathways in maintaining these in a previous issue. A more recent study focused on the phenomenon of confirmation bias, which is the disregarding of any information that contradicts one's beliefs. The authors used behavioural and neural modelling to assess any changes in the processing of information after we have made a decision, which may contribute to confirmation bias. They used magnetoencephalography brain scanning to monitor brain activity during an experiment consisting of a simple perceptual (visual) computer test in healthy participants. This approach, which limited cognitive and emotional input, allowed for neural information processing without motivational or social influences.

The participants were asked to make a decision about whether multiple dots on the computer screen were moving to the left or the right and then rate their level of confidence in their choice. They were subsequently presented with a clearer version of the test that would assist in making the correct choice and they were asked whether they would change their original decision. Brain activity, measured with magnetoencephalography during the test, enabled the researchers to assess the degree to which the participants processed this new information.

The brain activity of the participants who were less confident in their original choice showed that they used the new information in making their final decision. In contrast in those who were highly confident in their original choice their brains ignored the new information when it contradicted their choice but remained sensitive to information that confirmed it.

The researchers concluded that higher levels of confidence shape a selective neural gating for choice-consistent information, which reduces the ability to change an opinion in the light of new information. This confirmation bias, amplified by influences such as financial benefit, seeking or staying in power and other motivations in real life, can be recognised in some individuals in politics and society in general.

Rollwage M., Loosen A., Hauser T. U.,  (2020) Confidence drives a neural confirmation bias. Nature Communications, 11(1): 263410.1038/s41467-020-16278-6PMC725086732457308.

## Breathing to safety

Breathing is essential to our survival making sure our bodies are supplied with oxygen, but that is not all. According to a recent study, the rhythm of our breathing is important for our ability to remember and for our emotional judgement.

In animals such as rodents the rate of breathing drives slow oscillations as well as bursts of faster oscillations in the olfactory bulb and cortex and the breathing rhythms are thought to regulate cortical excitability and network interactions helping the shaping of smell, memory and behaviour.

A study in patients with epilepsy showed similar effects of breathing rhythm in humans. The authors were able to obtain electrophysiological data directly from the brains of patients undergoing surgery for intractable epilepsy. They found that the patients’ brain electrical signals fluctuated with breathing in areas that process emotions, memory and smell, namely the olfactory cortex and the limbic system, including the amygdala and the hippocampus. Interestingly, they also observed that the oscillations in these areas peaked when the patients breathed in through the nose but they dissipated when they breathed in through the mouth.

In addition to the electrophysiological tests, the researchers carried out behavioural experiments that examined the speed of emotional recognition and memory retrieval and found that breathing in enhances the speed of recognising a fear-producing situation and memory retrieval.

On the basis of their findings the researchers argued that rapid breathing that occurs in a panic state is physiologically beneficial to the brain as it results in a faster response to a dangerous stimulus.

Zelano C., Jiang H., Zhou G.,  (2016) Nasal respiration entrains human limbic oscillations and modulates cognitive function. Journal of Neuroscience, 36(49), 12448–672792796110.1523/JNEUROSCI.2586-16.2016PMC5148230.

## Is there a pain centre in the brain?

Pain is a common problem in humans and a major source of distress. Not surprisingly it has been extensively studied over many years and multiple areas of the brain and pathways have been identified but so far there has not been any evidence of a single centre in the brain that can switch pain off.

In a recent study in mice a research team from Duke University claim to have identified this key area in the brain. They noted that a subset of inhibitory neurons located in the central amygdala, are activated by general anaesthesia and they labelled these CeAga (Central Amygdala general anaesthesia). This location was a surprise to the researchers as the amygdala are associated with negative emotions and anxiety.

Giving a mild pain stimulus to the mice they found that these cells were connected and had an inhibitory input to at least 18 other centres in the brain, which are known to process sensory and emotional aspects of pain. Using optogenetics, a technology that uses light to activate a small cluster of cells in the brain, they were able to instantaneously terminate the experience of pain in the mice by stimulating the CeAga neurons. They also established that this neuronal centre can turn pain off but it cannot turn it on.

With further research these findings may open the door to the development of drugs that can specifically target this pain centre and hopefully achieve better pain control in humans.

Hua T., Chen B., Lu D,  (2020) General anaesthetics activate a potent central pain-suppression circuit in the amygdala. Nature Neuroscience, doi: 10.1038/s41593-020-0632-8PMC732961232424286.

Story Source: Materials provided by Duke University. Original written by Karl Leif Bates.

## What a headache!

Staying on the subject of pain, have you ever wondered why a pain in the head affects you more than the experience of pain elsewhere in the body? A study published 3 years ago found the answer to this question. They observed that the lateral parabrachial nucleus, which is essential to the affective pain circuit, is activated more strongly by painful stimulation of the face than the lower limbs.

The researchers developed a novel technology called CANE, which they used to identify and label noxious-stimulus-activated lateral parabrachial nucleus and they mapped comprehensively its anatomical input. This led to the discovery of a monosynaptic connection between the cranial sensory neurons and the lateral parabrachial nucleus pain sensitive neurons, which had not been known before. This finding indicates the presence of a direct connection between the craniofacial neurons to the brain's emotional centres and the amygdala. Whereas the body neurons have only indirect input.

When the researchers activated the direct craniofacial-to-lateral parabrachial nucleus projection using optogenetic technology they noted intense facial pain and its emotional effects were heightened while optogenetic silencing eased the facial pain. These findings lead the way to a better understanding of craniofacial pain intensity and associated emotion and hopefully the development of more effective treatments for very painful and distressing conditions such as migraine, cluster headaches, trigeminal neuralgia and others.

Rodriguez E., Sakurai K., Xu J.,  (2017) A craniofacial-specific monosynaptic circuit enables heightened affective pain. Nature Neuroscience, 20: 1734–432918420910.1038/s41593-017-0012-1PMC5819335.

